# Applying critical systems thinking to social prescribing: a relational model of stakeholder “buy-in”

**DOI:** 10.1186/s12913-020-05443-8

**Published:** 2020-06-24

**Authors:** Alison Fixsen, Helen Seers, Marie Polley, Jo Robins

**Affiliations:** 1 University of Westminster, School of Social Sciences (Psychology), 115 New Cavendish Street, London, W1W 6UW UK; 2 Shropshire Public Health, Shirehall, Abbey Foregate, SY2 6ND UK

**Keywords:** Social prescribing, Critical systems thinking, Service evaluation, Public health

## Abstract

**Background:**

Social prescribing (SP) allows health professionals to refer primary care patients toward health and wellbeing interventions and activities in the local community. Now widely implemented across the UK and adopted in other nations, questions arise concerning the modelling of present and future schemes, including challenges to full engagement encountered by stakeholders, which lie beyond the scope of traditional evaluations. Critical Systems Thinking (CST) allows for holistic analysis of fields where multiple stakeholders hold diverse interests and unequal power.

**Methods:**

We use CST to (a) critically examine a developing rural social prescribing scheme from multiple stakeholder perspectives and (b) present a relational model for local social prescribing schemes. Our fieldwork included 24 in-depth interviews, regular planning meetings with key stakeholders, and discussions with those involved with national and international SP landscaping. A modified grounded theory approach was used for the analysis, and to consider the core elements of social prescribing sustainability.

**Results:**

Our study confirms that local social prescribing schemes must operate with numerous stakeholder interests in mind, seeking to address real life social complexity and offer integrated solutions to multifaceted issues. Three main areas are discussed: holistic vision and boundary judgments; barriers and facilitators; relational issues and “emotional buy in”. Problems for staff include selecting suitable clients, feedback and technological issues and funding and evaluation pressures. Barriers for clients include health, transport and expense issues, also lack of prior information and GP involvement. Emotional “buy-in” emerged as essential for all stakeholders, but hard to sustain. Based on our findings we propose a positive relational model comprising shared vision, confidence and commitment; motivation and encouragement, support and wellbeing focus, collaborative relationships, communication and feedback, access to information /resources, learning in and from action, with emotional “buy-in” at its heart.

**Conclusion:**

Those implementing social prescribing in different localities inevitably face hard choices about what and whom to include. Research on the sustainability of social prescribing remains limited, studies are required to ascertain which “holistic” models of social prescribing work best for which communities, who are the main beneficiaries of these approaches and how “buy-in” is best sustained.

## Background

Social prescribing is rapidly growing in scale in the UK and attracting international interest [1]. Its use of link workers to refer people toward health and wellbeing interventions and activities in the community presents a real alternative to traditional medical solutions to illness [2] that have failed to halt the rising tide of chronic mental and physical morbidity. To date the research into social prescribing has largely focused on outcomes, but its relational dimension is also a rich and complex field of study. Pertinent questions remain concerning the operation of local social prescribing schemes, including their ability to allocate resources to those who most need them and to ensure fair and meaningful participation of all stakeholders involved in the planning process, which traditional evaluation methods are unlikely to answer. With health and social care resourcing- both financial and human- at crisis level the ability of local social prescribing schemes to locate, recruit and activate low-agency clients, and to direct them toward suitable community-based activities and interventions in a sustainable manner, is crucial.

Despite extensive research into the outcomes of social prescribing, critical discussion around relational theories associated with social prescribing is lacking which, as social prescribing schemes grow in scale, is a matter of urgency. Systems methodologies have been widely applied in health contexts research [3] and critical systems thinking (CST) specifically encourages a holistic analysis of complex social problems and interventions [4], making it of particular relevance to social prescribing.

In this article, we use CST to critically examine a developing social prescribing scheme from multiple stakeholder perspectives and present a relational model for local social prescribing schemes. We begin with an overview of social prescribing.

### Social prescribing in the UK

Social prescribing seeks to address the wider determinants of health by expanding the range of non-medical options, where health problems are related to socioeconomic and psychosocial realities [3]. Using a collaborative approach, it provides healthcare professionals with a referral option towards various pro-health inventions in the community. There are many models of social prescribing [4], however a typical pathway is that the public health and social care provider refers a client or patient to a social prescribing “link worker” who engages them in a conversation (typically for 30–60 min) in order to co-produce a personalized solution or “social prescription.” The link worker then refers the client to a “recognized” local voluntary social and community enterprise (VCSE) offering a suitable form of social and psychological support, which may include physical activities, art classes, gardening, or welfare, work and legal advice [5]. In complex cases, the individual may need several meetings with a link worker to share their whole narrative or may be referred back to the health system if they have not been receiving the care they need. Earlier versions of social prescribing services were delivered by local neighborhood and community organizations and by volunteers working with single GPs, and were not part of mainstream statutory delivery [6]. NHS England now includes social prescribing as one of the six pillars of its Universal Personalised Care Strategy [7], specifically providing ringfenced funding for all Primary Care Networks to fund a link worker by 2021 [8]. At one time an option, social prescribing link workers will soon be a feature of all UK GP practices.

The general case for social prescribing seems strong. With its focus on the bio-psycho-social model of illness, social prescribing can address problems preceding or accompanying stress on health and wellbeing from multiple angles [9, 10]. From a services and resources perspective, the widespread use of social prescribing has the potential to (a) reduce costs related to multi-morbidities [11], (b) reduce demand on primary, secondary and social care services [6] and (c) relieve pressure on GPs working long hours, dealing with large patient caseloads and treating patients with complex needs [12]. Determining the overall “value” of social prescribing versus other interventions has proved difficult however, as many of its returns are of the social and emotional, rather than any economic calculable variety [12, 13].

Much qualitative data on social prescribing supports the notion of social and emotional returns such as adding “meaning to medicine” [14, 15] and creating or strengthening relationships within local communities [16]. Through their collaborative work with the Social Prescribing Network, Polley et al. [17] identified key operational and relational ingredients of social prescribing which include: collaborative relationships with people in different sectors, funding commitment, good service infrastructure, a clear referral process, skilled and supported link workers, patient centeredness and crucially, buy-in from professionals (GPs/ health care workers) and their patients. Central to these ingredients are integration, communication and feedback, and underpinning them research and evaluation [17], p.14).

### Critical systems thinking (CST)

With its roots in antiquity, “systems thinking,” as a branch of science, emerged in the mid-twentieth century through a critique of reductionism and its predilection for studying phenomena as distinct rather than whole entities [18, 19]. Contemporary systems thinkers operate somewhere on a continuum from “hard” to “soft,” with relational thinking- recognizing how people’s actions and the processes that steer them energize or drain the whole system and those within it- seen as key to organizational transformation [20, 21]. Use of soft systems thinking/methods (e.g. team building, learning and feedback loops) has now been widely promoted for strengthening healthcare systems [20–22]. One example, Bell and Christina’s (2006) study, concerns the application of soft systems modelling (SSM) [23] in the integrated planning of complex projects (in this case workforce re-modelling) within the NHS in Shropshire and Staffordshire, UK [24]. The authors identify three ways in which SSM could be applied to provide outcomes essential for a successful project: a rich picture of what is, a root definition of what could be and an activity plan or conceptual model of how to get there [24].

Different systems approaches emphasize different aspects of complexity. First formulated by Churchman [25], the term critical systems thinking (CST) is used here to describe a meta-approach that seeks to draw together the critical elements of SSM with broader philosophical and social theory in a creative and flexible way [19]. Philosophically it focuses on issues such as emergence (change, dynamics), interrelatedness (holism), unpredictability (limits to knowledge and foresight), and power issues such as inequalities and marginalization in the construction of social reality and in action [19, 24, 26, 27]. Firmly opposing functionalist approaches that imply regularity, causality and determinism in human behavior, CST recognizes that certain ideas and groups will be excluded from decision making and allocation processes. Choices or “boundary judgments” [25–28] are made about what to include in any particular vision or plan and who will be the beneficiaries of these actions, which from a CST perspective should be based on equitability and sustainability [19, 28]. In CST, the focus on emergence, interrelatedness and sustainability go hand in hand, local/organizational expansion should therefore proceed synergistically with consideration for the future society/planet [19, 28, 29]. For Bell and Morse [29], a holistic approach to sustainability requires involving local communities (not just experts) in decision making from inception, including long-term objectives, seeking long term funding and addressing spiritual, ethical and cultural concerns (p.18). Aims of social prescribing such as use of local people and assets; reduction in prescribed medication (especially antibiotics and environmentally polluting drugs), ambulance callouts and hospital admissions; and promoting green energy pursuits all seem positive sustainability steps. Even so, complex questions remain about the sustainability of social prescribing, which a CST approach can help to answer.

Despite their common roots and interests (holism, question taken-for-granted traditions, seeking of social and individual transformation via more equitable processes [19, 20, 26, 27], CST has yet to find a place in the social prescribing literature. As social prescribing expands, CST can be used to formulate critical, challenging questions about present and future. Why, for instance, is one type of intervention working for some people and not others? Could some models of social prescribing increase rather than lessen health and social inequalities in some areas, and if so, what can be done to encourage the distribution of social prescribing resources to those most in need of them? In social prescribing these are vital issues as they relate to phenomena such as social determinants, health equity and marginalization (of stakeholders and health and social issues).

Finally, while relationality is a key concept for both CST [18, 19, 30] and social prescribing [3, 9, 10] we detected a lack of exploration of the emotions/feeling dimension in both sets of literature. A sociology of emotion would argue that feelings (passions and enthusiasms, doubts, contradictions and conflicts) are the mainstay of human life and a key influence on “rational” decision making [31, 32] which impact the sustainability of any project. Thus, a key aspect of our work is to expand the relational focus of social prescribing and to explain the importance of “emotional buy-in.”

## Methods

### Study context

The study we describe in this paper forms part of a service evaluation on a social prescribing scheme that took place between January 2018 and July 2019 in the rural county of Shropshire, West Midlands, UK. The scheme sits within a wider Shropshire health and wellbeing strategy aimed promoting health and reducing health inequalities within this community [33]. Public health issues in Shropshire causing high levels of demand on health and social care expenditure include cardiovascular disease, diabetes and prediabetes, musculoskeletal disease, respiratory disease and falls in older people. The rurality poses its own challenges, such as food poverty [34], social isolation issues, difficulties accessing services and adequate response times for the emergency services.

### Aims and study questions

This study aims to explore one rural UK social prescribing scheme while under development from a critical systems perspective. Key CST questions we posed when carrying out our qualitative study were: how have the Shropshire social prescribing team gone about co-producing initiatives which address their key objectives? What boundary judgements were made and why? And what learning took place in the process?

### Procedure

The qualitative data described in this article were part of a longitudinal service evaluation commissioned by Shropshire Council in 2017, conducted by the first three authors. The third author, a core member of the Shropshire team, provided background of the service. Ethical approval for all steps of the study were given by the University of Westminster Research Ethics Committee. The evaluation had two elements, a quantitative service evaluation involving 135 social prescribing service users, and a qualitative systemic enquiry of the developing scheme, based largely on stakeholder interviews and informal meetings and discussions. In practice these different strands of the evaluation informed the other. For the service evaluation, two specific cohorts were identified for referral to the social prescribing scheme: (1) patients with a cardiovascular disease (CVD) risk score of 10% from two participating GP practices and (2) individuals identified as at risk of loneliness or social isolation, referred opportunistically by GPs, GP practice staff and external organizations (e.g. Department of Work and Pensions (DWP), Adult Social Care). Those who agreed to take part in the evaluation were asked to complete a series of questionnaires at the first consultation and at the 3-month follow-up. Once the collection of health measurement data had reached a certain level, a phased process of qualitative data gathering commenced (see Table 1).

**Table 1:** Table of participants. This table provides a breakdown of the interviews and their dates in three columns. Pseudonyms or numerical identifiers were used for all participants. Key staff interviews were face-to-face, all other interviews were conducted by phone. A table key explains the key participant characteristics

Key Staff*	Year	Other stakeholders*	Year	Service users	code	Year	Ref
Link worker (Carol)	2018	Practice manager (Keith)	2018	Female	000	2018	CVD
Public health manager (Joan)	2018	DWP Disability Employment Advisor (Sue)	2019	Female	001	2018	CVD
Project Manager (Ken)	2018	DWP Employment Advisor (Pat)	2019	Female	002	2018	CVD
IT manager (Chris)	2018	DWP Employment Advisor (Ann)	2019	Male	003	2018	CVD
Service manager (Kath)	2018	Adult social care worker (Jill)	2019	Male	004	2018	CVD
Public Health consultant (Elise)	2018	Adult social care worker (Mel)	2019	Male	005	2018	CVD
Link worker (Emily)	2019	GP (Nick)	2019	Female	006	2019	Other
		GP (Liz)	2019	Female	007	2019	Other
				Male	008	2019	MH
				Female	009	2019	Other

Key: Participant Characteristics

• Link worker = social prescribing link worker

• DWP = Department of Work and Pensions

• Code = anonymous code for paper only

• Ref = referrals

• Service user = a person who has met with a social prescribing link worker

• CVD = service user is on a CVD register

• Other = service user has been identified or self identifies as having health issues related to life style, weight.

• MH = patient has been identified as having mental health issues

Note: Key staff interviews were face to face, all other interviews were conducted by phone

### Qualitative arm

A case study method was used for the qualitative arm to provide rich description from a wide range of stakeholders [24, 35]. Twenty-four people were interviewed by the first author, either face-to-face or over the phone. In Phase 1, 6 staff members directly involved in the designing, implementation and running of the scheme and 6 people from the CVD (cardio-vascular disease) risk register who had met with a link worker on at least one occasion were individually interviewed. Around 6 months into data collection, two reports (an evaluation and qualitative analysis) were presented for a round table discussion with external stakeholders. At the end of Phase 1 (December 2018), the research team submitted their interim report, with recommendations. In Phase 2, 4 non-CVD service users and 8 external stakeholders from organizations referring into the scheme were contacted and interviewed over the phone. Additional to these interviews were frequent discussions with team members concerning the progress and setbacks that they were encountering. Due to time and budget constraints, members of voluntary and community organizations were not interviewed, but were included in wider conversations at steering group meetings. A final report incorporating quantitative and qualitative data and analysis, along with recommendations and conclusions, was submitted to the service in July 2019.

### Interviews

We designed separate semi-structured interview guides for professional and service users, using open-ended questions and prompts to direct the conversation, while allowing participants to relate their stories and for unanticipated issues to emerge (see Supplementary files: Service user interview schedule and Professionals interview schedule). Prior to all interviews, participants were sent copies of the participant information sheet to read and/or had the study clearly explained to them over the phone. All but one participant then consented to take part (one service user felt they were not up to doing it). Interviews were held at the convenience of the participant, and the dialogue was digitally recorded. All participants were debriefed and thanked after interview. Interview data was transcribed by an agency and stored securely as password protected files on a password-protected computer. Table 1 provides a breakdown of the interviews and their dates in three columns. Pseudonyms or numerical identifiers were used for all participants, no real names were used.

### Analysis

**Fig. 1:**
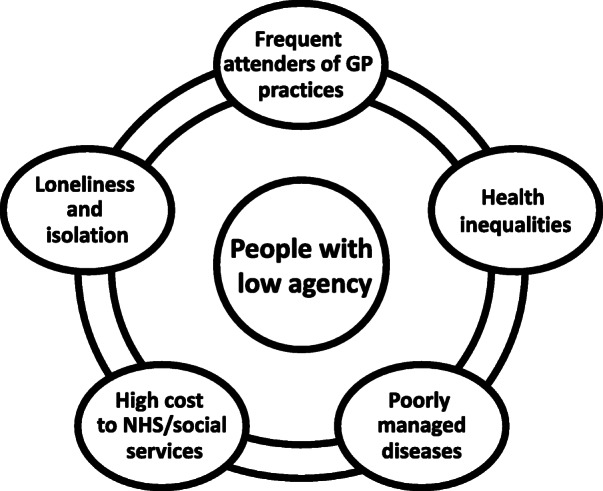
*Diagram 1: Team vision map*. This concept map was created to identify the “team vision” emerging from the 6 core stakeholder interviews. It identifies the target areas of the pilot scheme as: loneliness and isolation; frequent attenders at GP practices; health inequalities; poorly managed diseases; high cost to NHS and social services, all of which pivot around people with low agency. The findings from this first part of the study were presented at a meeting of stakeholders including GPs and practice managers for feedback and also formed part of the Phase 1 interim report

Using modified grounded theory, data was explored from multiple angles and developed over time [36]. As ideas were generated, they were discussed with research team members social prescribing stakeholders within and outside this particular scheme, to establish their coherence. In the first stages of analysis, data from different interviews and stakeholder discussions were considered as separate elements by the first author, a qualitative research consultant, with no prior connections with the scheme. A concept map (diagram 1) was created to identify the “team vision” emerging from the 6 core stakeholder interviews which identifies the target areas of the pilot scheme: loneliness and isolation; frequent attenders at GP practices; health inequalities; poorly managed diseases; high cost to NHS and social services, all of which pivot around people with low agency. The findings from this first part of the study were presented at a meeting of stakeholders including GPs and practice managers for feedback and also formed part of the Phase 1 interim report (Fig. 1).

### Coding process

Data was coded using a modified constant comparison approach, inspecting and comparing all data and fragments arising in a given case and moving from a larger to more compact data set [37]. By repeatedly listening to the digital recordings and reading transcripts of interviews, author one familiarised herself with all the data covering the full range of themes. In addition to manual memos and coding, all interviews were coded using NVivo software (See supplementary file: Word frequency cloud stakeholder interviews). At different stages of data analysis, emerging codes and themes were discussed with author 2. As concepts emerged or were discarded, further inductive coding (at open and axial levels) was performed and linked to existing theory in the literature, constantly referring back and forth between the data and evolving theory, to ensure the latter remained grounded in the experiences and narratives of my research participants [36]. Here, open coding refers to breaking down, examining, comparing and conceptualising of data, and axial coding to the putting back together of data in new ways to make sense of categories [36]. NVivo was also used for extensive query and text searches, to analyse different sections of the data in various ways and for safe storage of transcript data and other information relating to the project. As interviews were done over time, it was simple and logical to choose initial codes from early interviews, and develop theory, to later modify and expand them as different information emerged. Finally, all accounts were amalgamated and analyzed, to present the themed narrative and discussion presented in this article.

## Results

Our study highlighted multiple and complex relational and organizational factors associated with the development of social prescribing in Shropshire. Here we focus on four main themes: the vision behind the project; team learning (in and from action); complexity of the link worker role; and stakeholder and service user “buy-in.”

### The vision

The roots of the Shropshire social prescribing scheme lie in the countywide health and wellbeing strategy and its shift in focus from “fixing disease” towards promoting and maintaining health by “working collectively to identify and test out solutions [36]. The core team (Help2change, Public Health England) driving the scheme spoke passionately about their commitment to this new venture and its potential outcomes. Ken, a former GP and a key architect of the scheme, described his vision of long-term transformation and of leaving a *“legacy that is going to be around in 20 years’ time*.” The task was to create a sustainable, investible service, while allowing people to have holistic care via a multitude of services. Resources were limited however, and the team had needed to make strategic choices about whom to priorities. The Shropshire service had chosen to target people with “*low agency*,” that is those less likely to take up offers of signposting without the support of a link worker, and those who were on a *“really unhealthy journey”* and were *“costing the NHS millions.”*

With the UK GP workforce in crisis [38], a big *“driver”* behind setting up the Shropshire scheme had been to relieve local GP workloads. Social prescribing, according to GP Liz, was like saying, *“you don’t need to come here, you don’t need the pills, there are other things that you can be doing before you get to that point.”* For a GP, having someone coming in with multiple issues when you didn’t physically have the time to tackle them was frustrating. GP Nick described the type of person and issues he might refer on to social prescribing:

“*[They] will be the people with low level depression, anxiety, loneliness, isolation, people with chronic disease management problems to try and encourage them to take ownership in certain cases*. *.*. *frequent attenders with perhaps non confirmed diagnosis.”*

Those involved in delivering social prescribing identified two elements that were crucial to its success; the role of the link worker and the presence of a *“thriving”* community sector in the locality. It was in meetings with the link worker that individuals could establish their needs and, through a process of co-production, put a plan into place and refer people to appropriate agencies. VCSE services could also benefit from the formal referral and feedback system, in terms of securing future funding. It was important to treat stakeholders in these organizations as equal partners, and *“not dictate to them.”*

### Learning in and from action

Fundamental to a “learning organization” is unconstrained discussion and exchange of issues faced and their prioritization towards organizational change [39]. The initial step (November 2016) had been to scope the established UK social prescribing services (e.g. Rotherham, Doncaster, South Gloucestershire, Bow). This was followed by discussions and visionary events with stakeholders from the voluntary sector, the local hospice, the Clinical Commissioning Group (CCG), Help2change and council directors to work out where social prescribing might fit in, what it could support and what resources were already in existence. Two models were employed to develop the scheme; an agile change model to ensure that *“operational”* team members were working collaboratively and a “Health and Care Large Scale Change Model” for the development of a common purpose. All the above procedures were carefully documented to ensure that “the team, as it develops, has learnt as well.”

What Ackoff [40] refers to as “formulating the mess” (where mess is the consequence of the system’s current state of affairs) [41] is a means of helping people to work with (rather than counter to) the organizational muddles they are in and which would continue to hinder them were things to remain as before [26]. Part of the core team’s learning journey had been recognizing and learning from the operational and relational problems they had encountered in setting up the service with a small, mostly part-time team and limited monetary resources. The team’s decision to conduct an evaluation virtually in tandem with its early development placed them under extreme pressure to secure results that would bring in the second stage funding needed to *“scale up [social prescribing] across the county*” (Joan). Technical hitches, such as working out the best way to input data, securing a license for one of the questionnaires and initial problems with service user recruitment each slowed the process down. Despite these setbacks, the energy and commitment of the Shropshire team, and their frequent meetings and communications with the researchers, enabled important deadlines to be met, suggestions from the Phase I report to be followed up and the final service evaluation report be completed on schedule. Two years on, the scheme is now fully operational and merits the description of countywide social prescribing service, with link workers positioned in a number of GP surgeries.

### Link worker role

Research done in areas of the “people” sector indicates that, even where self-chosen, intensive face-to-face work can be stressful and emotionally exhausting [42, 43]. While the link workers we interviewed spoke enthusiastically about their work, the complexity of the role could be hard to navigate. Frequently they were dealing with multiple issues, so it was a case of deciding which to tackle first; *“Do we try and boost your confidence … or go for something that helps with depression and anxiety?”* (Link Worker 1). Link Worker 2 described her role as *“not for the faint hearted.”* Frequently clients had a lot of things going on in their lives (e.g. financial problems, domestic problems) and some arrived emotional, angry or stressed, or became so when they did not like a survey question or a suggested intervention. Link Worker 1, who was involved in the training of new link workers, explained that the new recruits had counselling or health care backgrounds and received training in motivational interviewing and behavioral change, as well as supervision. Still, Link Worker 2 felt that additional courses on thorny issues such as anxiety and depression, abuse and addiction could assist link workers in this emotionally complex work.

### Stakeholder “buy-in”

The term “buy-in” has been used in the social prescribing [44] and other management and health care literature [45, 46] to describe a complex process by which different stakeholders negotiate their commitment to processes or actions, in which they are directly, or indirectly, involved. External stakeholders in our study, including GPs, were largely chosen because of their experience in referring clients or patients to a social prescribing link worker. Most of those interviewed were very positive about the scheme and were keen for it to continue and grow, e.g. *“[There was] lots of enthusiasm from the team - they were really positive, and it has been nice actually … working with people outside of the mainstream NHS”* (Nick). Nevertheless, they were aware of initial suspicion and even rivalry on the part of some colleagues, for example a few community care coordinators (CCCs) (who worked predominantly with the fail and elderly) had concerns that the link worker might duplicate, or even usurp, their own role. Both the GPs and practice manager knew of doctors who resisted any directives coming from authorities other than the CCG and who had doubts about the skills and training of the link workers. Even where staff within practices were more engaged, *“you have to constantly communicate, and recommunicate, and remind practices and staff it [social prescribing] is there”* (Liz). Over time and with more involvement resistance has lessened; more GPs and GP practices have now joined the scheme and at least some CCCs are now referring clients into the scheme.

The limited resources of most social prescribing schemes and unpredictability of individual outcomes mean that even those with quite high levels of emotional investment in social prescribing face dilemmas when having to decide which clients or patients should be referred. GP Nick explained how, while some patients really latched onto social prescribing, others- *“don’t want to move out of the medical model because they’re still not [ready]”* (Nick). For practice staff, having to deal with the initial problems and setbacks of the pilot, along with complaints from certain patients confused or affronted at being contacted had created some feelings of frustration. It was taking time for a link worker to be accepted as part of a practice landscape, and in the light of new General Data Protection Regulation (GDPR) legislation, storage of data and link worker access to patient notes were still on-going issues to be negotiated.

As more agents, with differing views and values, get involved in a project, so buy-in becomes more critical and yet harder to maintain. Under the Shropshire scheme professionals working in allied organizations such as the DWP and Adult Social Care were also encouraged to refer suitable clients to a link worker. Staff interviewed from these sectors agreed that, in principle, social prescribing was a great idea, but motivating people in an area of high deprivation was a big challenge:

“*We want it to work but [it’s a big task]*. *.*. *the North West of Shropshire is one of the most deprived areas of the county, there are a lot of people who*. *.*. *have got compromised health or disabilities because of their level of income opportunities, health, there’s a lot of people that don’t work, smoke heavily, have got poor diets, overuse of alcohol, so [this is] definitely one of the right areas to target.” Ann*

Typical referrals from the DWP had included frequent attendees at GP surgeries, and the young and isolated, and some were, *“second and third generation customers- mum and dad [or siblings] have never worked”* (Jill). Referrals had, however, dropped off since the link worker no longer came regularly to their premises, reinforcing the idea that the visible presence of the link worker on the premises promotes buy-in from allied stakeholders.

Those working in Adult Social Care also had success stories, although some attempts to refer clients to link workers have proved unsuccessful. Many of their clients were elderly and housebound, so were unable to get to GP practices and were already visited by CCCs. When asked what might encourage staff to refer more people to a link worker, more feedback from link workers regarding the progress of people they had referred into the scheme, and *“working more closely with our health colleagues- being part of that promotion and preventative role,”* were advocated. These responses highlight the desirability of early collaboration and greater involvement in the social prescribing process of allied stakeholder buy-in.

### Service user “buy-in”

Social prescribing provides a means of referring and signposting people to activities of which they may be unaware or, for various reasons, may have discounted. Over the course of a year, we interviewed people who had seen a link worker on at least one occasion. As patients in the lonely and isolated category had proved hard to recruit for the evaluation, the majority of those interviewed had been referred through the CVD register. All participants in our sample recalled having seen a GP or health professional at some point with a personal health concern, such as needing to lose weight or reduce high BP (blood pressure) or cholesterol, but none recalled a discussion with their GP around social prescribing; e.g. *“I certainly don’t recollect being pointed in that direction …*. *I was surprised when I received the letter.”* The first time most had heard of the scheme was when contacted by letter or referred by another health professional, such as a physiotherapist. Some had been quick to take up the offer of the service, others had been promoted to do so by a follow up call, *“if they hadn’t persisted, I would have forgotten about it.”*

As a whole, the users were interviewed appreciated the person-centered approach of the service and contrasted this with the brief time afforded by GP appointment slots:

*“How often do you get offered an hour’s chat about a particular problem with a doctor in the medical center? You don’t, and I have to say that was really quite an incentive.”* (002)

Link workers were also described in a positive light (“*very helpful,” “supportive,” “listened carefully and came up with good suggestions”*). There were, however, a few service users who were disappointed when a link worker changed or would have liked a larger number of visits to the link worker.

Those who followed the suggested intervention or treatment had different outcomes to report. Some had found them very helpful for attaining personal health goals. e.g., *“I’ve lost one stone three pounds in six months, which is very heart-warming”; “I’ve given up (smoking)”; “My BP (blood pressure) is down today, so something is workin*g.” Others were already pursing their own health or fitness regime before seeing the link worker; “*She [the link worker] said ‘you are already going everything you can’.”* Social prescribing is not conceived of as a long-term intervention, however two service users expressed disappointment that they had finished a program (in this case, a swimming and managing diabetes program) before they had gained much benefit, e.g.*, “I really enjoyed it, felt so much better for it, but it wasn’t enough.”* Others mentioned the practicalities of travel, which in a rural county could mean quite a long journey, and the expense of attending classes. Even with at a reduced cost, gym membership seemed too much for one participant to afford on a regular basis.

Suggestions were also made concerning ways to enhance the service and its evaluation processes. These included providing more information about social prescribing in GP practices, filling out questionnaires before or after the meeting and the allocation of more funding to the voluntary sector; “*As ever in the health service and the council, it’s about money isn’t it?”* Several service users wanted to see GPs engaging more with social prescribing; “*I think, if you’re talking about people’s overall general welfare and health then you need to get the GPs more involved.”* Whatever their personal experiences or criticisms, service users as a body believed social prescribing and its person-centered ethos to be a good thing and said they would recommend it to others; *“I’d say do it, without a doubt.”*

### Toward a model of sustainable social prescribing

**Fig. 2:**
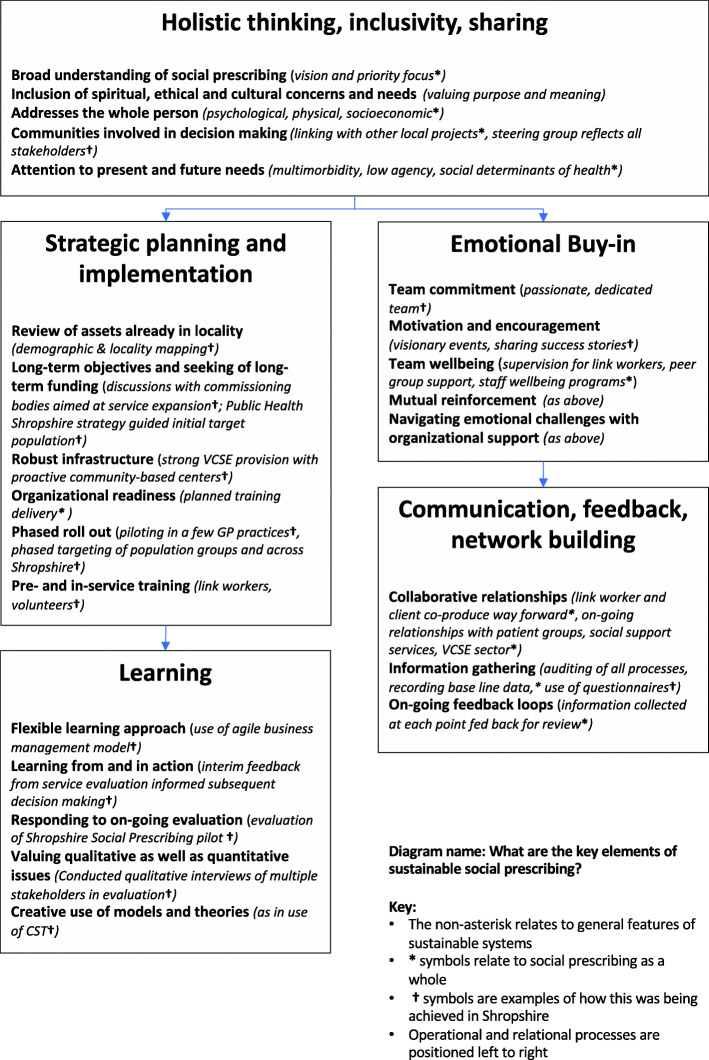
*Diagram 2: What are the key elements of sustainable social prescribing?*. The diagram is essentially a flow chart that represents what we regard as core ingredients of system sustainability, as they relate to social prescribing in general (**†**), and to examples from our study (*). The box at the top of the chart concerns ‘Holistic thinking, inclusivity, sharing’. ‘Operational and relational processes’ are positioned left to right. Boxes on the left concern ‘Strategic planning and implementation’ and ‘Learning’. Boxes on the right concern ‘Emotional Buy-in’ and ‘Communication, feedback, network building’*.* Key systems literature informing this diagram include Churchman [24], Bell and Morse [29], WHO Alliance for Health Policy and Systems [20] and Flood and Finnestrand [19]. Key social prescribing sources include Polley et al. [17] and NHS Scotland [47]. In collating the flowchart we identified a unique driver of social prescribing, emotional buy-in

From our study findings and the literature we have constructed the following diagram which represents what we regard as core ingredients of system sustainability, as they relate to social prescribing in general (**†**), and to examples from our study (*). Key systems literature informing this diagram include Churchman [24], Bell and Morse [29], WHO Alliance for Health Policy and Systems [20] and Flood and Finnestrand [19]. Key social prescribing sources include Polley et al. [17] and NHS Scotland [47]. In collating the flowchart we identified a unique driver of social prescribing – emotional buy-in – and in process have highlighted a potential gap in existing CST relational modelling (Fig. 2).

## Discussion

A systems approach can offer health care organizations ideas about how to operate more effectively and sustainably in complex, real-world settings [18–20, 22]. We have used CST to examine the vision, aspirations and boundary judgments of a local social prescribing scheme as it emerged, transitioned and expanded from demonstrator sight to countywide service. Our findings confirm the operational and relational complexity facing the social prescribing team in Shropshire and their collaborative learning journey as the scheme progressed. Having in place a phased roll-out plan and making use of existing local assets both helped to keep the project on track and sustainable, while evaluating the service has helped to secure longer term funding. We now reflect on the following points in the light of the wider systems and social prescribing literature; holistic vision and boundary judgments; barriers and facilitators; relational issues and emotional buy-in.

### Holistic vision v boundary judgment

A shared vision – collaborative development of a shared mental model realizing a common sense of purpose- [19] lies at the heart of a systems dynamics approach. Many advocates of social prescribing favor a holistic approach, generally in the sense of applying a bio-psycho-social model, rather than a biomedical (reductionist) model, of health and illness [2]. The “holistic” relationship between link workers and individuals is seen as particularly important, both for supporting individuals in behavior change and addressing entrenched social issues [44]. “Holistic” social prescribing projects (arts, fitness, music etc.) identified in one Bristol study that emerged from organic partnerships between GPs and their local third sector partners had, at 3 months, increased wellbeing, led to better friendships and reduced GP attendance rates in participants [13]. Research on the impact of social prescribing on use of primary healthcare resources remains limited, however [47, 48]; further evaluation studies are required to ascertain which “holistic” models of social prescribing work best for which communities and who are the main beneficiaries of these approaches.

CST informs us that practitioners make boundary judgements about what and who should be included in any venture and who will be the beneficiaries of any proposed action and which stakeholders will not (yet) be represented [19, 49]. These boundary judgements emerge and change with socio-political structures, with choices informed at individual, local and/or central level. Social prescribing schemes, both within and outside the UK, work from and with different models, for example some schemes are largely community-led with link workers located in community venues [5, 50], others have link worker programs attached to GP practices and more centrally financed and monitored [51]. The Shropshire scheme has been flexible in having a link worker program which operates out of both GP practices, community centers and venues such as the DWP. Non-medical as well as health care staff could refer people to link workers. Unlike some schemes [51] however, not everyone can (as yet) be referred or can self-refer into the scheme. In summary, while social prescribing is envisaged in the NHS Long-Term Plan as universally accessible, those implementing social prescribing in different localities will inevitably face hard choices about who to include in both decision making and provision, particularly in their early days.

### Barriers and facilitators

Various studies of social prescribing have focused on the enabling and obstructing factors impacting particular or multiple schemes. For example, Pecheny et al’s [52] meta-analysis of eight UK social prescribing schemes identified a range of facilitators/barriers to implementation and delivery, including legal agreements, leadership, management and organization issues, staff turnover and engagement, relationships and communication and local infrastructure. Reported barriers to uptake and adherence in service users in Peschney et al’s [53] qualitative study included fear of stigma of psychosocial problems, patient expectations and the short-term nature of the program; other studies have pointed to different access barriers [54, 55]. In our study, fear of stigma was not mentioned as a problem, whereas short-term programs were, along with barriers more specifically associated with the population group and locality.

Complex multimorbidity is known to be far more common in deprived areas [54]; the social prescribing service in Shropshire operates in an area in which food poverty, transportation difficulties, high unemployment and multimorbidity all feature [34]. Recruiting people in the lonely and isolated category had proved particularly difficult in the early phase of this study, however by working with allied services such as the CCCs, DWP and Adult Social Care workers, and potentially fire and emergency services and others with local knowledge, these more vulnerable and harder to reach groups may be better included in future. More research is needed that helps policy makers and those developing and running schemes understand processes through which people with complex problems and low agency are empowered to access and use social prescribing services in particular areas [54], for example through wider use of digital technology [55].

### Relational dimension and emotional buy-in

CST seeks to open up debate over relational issues that impact on the lives of people in organizations to consider how their responses influence the way things are now and how they might be in the future [18, 19]. Previous qualitative studies have emphasized the importance of the relational dimension of social prescribing, however much data collection has concerned specific stakeholder relationships such as the support/signposting offered by link worker to service user [14], the collaborative relationship between community link worker and community organization representatives [4], and the peer relationships developed in community based settings [13]. Our study has taken a broad perspective of relationship building in social prescribing, and emphasizes the part played by emotional buy-in in the process.

One factor affecting buy-in from health and social care providers (including link workers) emerging from this and other studies is that of emotional exhaustion [43]. GPs and other health and social care providers in areas of high deprivation are frequently overstretched and stressed [38, 56], hence the high importance of and staff wellbeing programs, peer group support and provision of regular supervision for those working in such localities [3, 38, 47]. In theory there is no reason why all GPs should “buy-in” to the ethos of social prescribing, nevertheless all stakeholders in our study felt that GPs should be involved in and informed about social prescribing, findings which concur with other real-life studies of social prescribing at local level [44].

Service users with complex needs, who receive multiple appointments and visit health and social different providers, can also suffer from what might be termed “system exhaustion.” Studies suggest that clear, consistent messages from health providers can inspire confidence in patients, while involving them in decision making was more satisfying for both parties [57]. Much more could be said concerning emotional buy-in and the community sector, however this is the topic of other studies [16].

**Fig. 3:**
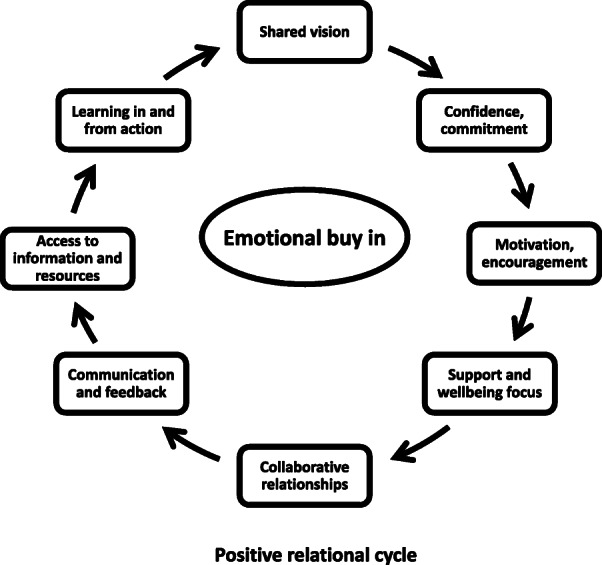
*Diagram 3: Positive relational cycle*. This diagram illustrates a positive relational cycle which would contribute to the success and sustainability of local social prescribing programs. and similar schemes. The elements identified are: ‘shared vision’; ‘confidence and commitment’; ‘motivation and encouragement’; ‘support and wellbeing focus’; ‘collaborative relationships’; ‘communication and feedback’; ‘access to information and resources’; ‘learning in and from action’. Boxes on the left represent the more operational components, those on the right are more relational, while emotional buy-in is located at the “heart” of both

Emotional buy-in, we suggest, rests on four foundations; that people regard the system as worthy *(confidence, passion, commitment);* that people are sufficiently well informed concerning the purpose of the system and their role in it even as the system develops and changes *(clear vision, communication, feedback, feedback loops);* that people are able to engage with the system and learn in and through it (*audit trail, action learning loops);* and crucially, that people feel sufficiently valued and rewarded for the efforts they put into the system *(motivation, encouragement, support/wellbeing focus).* This “positive relational cycle” is illustrated in the diagram below. Boxes on the left represent more operational components, those on the right are more relational, while emotional buy-in is located at the “heart” of both (Fig. 3).

## Conclusions

Our findings indicate that social prescribing schemes, such as the one in our study, operate with many interests in mind. They have to constantly balance the needs of different agents to ensure that no part of the system is hindering the progress of another. Using a systems approach has enabled us to reflect upon and share the experiences and concerns of our study participants, but also to look at issues such as sustainability, participation and disadvantage in different ways, such as through examining emotional buy-in. Our work confirms the critical importance of focusing on inter relational issues and “co-production” between health care, other professions, and patients/service-users within social prescribing and how best to achieve these ends deserves further consideration. In conclusion, there are lessons that health researchers can learn by engaging directly with the core work of the systems thinking community. There are also insights from real-world studies which those with an understanding of emotion in social interaction can share with other systems thinkers, and we welcome more interdisciplinary collaborations between persons with serious concerns about human welfare within medicine and social science.

### Study limitations and future research

This was a small study within a rural location, possibly limiting its application to urban settings. The problems we have discussed- including the difficulty of achieving a “vision” whilst taking into account the personal and interdisciplinary issues that distract and divert energy and commitment within and around organizational services - are nevertheless general to health and social care systems. One study limitation is failure to interview members of the voluntary and community sector, however this has been subject of another study by the authors [16]. A further limitation is absence of certain disadvantaged/minority population groups from the discussion, something that we are now pursuing in a further study. In addition, lessons from the present crisis situation from COVID-19 concerning enforced social isolation suggest that novel ways to recruit, support and deliver social health and wellbeing activities may need to be considered of all future social prescribing planning. Finally, while still in the development stage, we believe our sustainability chart and relational model will be useful for building and possibly evaluating future health programs that aspire to more holistic and collaborative ways of working.

## Supplementary information

**Additional file 1.** Service user interview schedule: List of open questions used as a guide during the semi-structured interviews with service users for the qualitative arm of the service evaluation Shropshire social prescribing project.

**Additional file 2.** Professionals interview schedule: List of open questions used as a guide during the semi-structured interviews with professionals for the qualitative arm of the service evaluation Shropshire social prescribing project.

**Additional file 3.** Word frequency cloud core stakeholder interviews: created through NVivo software, this diagram illustrates the frequency with which participants who were core team members used a word or phrase during the semi-structured interview. The word at the “heart” of the cloud is “people,” pointing to the importance placed on the relational element of social prescribing.

## Data Availability

The full qualitative dataset analyzed in this study is not publicly available for privacy and data protection reasons.

## Abbreviations

BP: Blood pressure
CCG: Clinical commissioning group
CST: Critical systems thinking
CVD: Cardio-vascular disease
DWP: Department of Work and Pensions
GDPR: General data protection regulation
GP: General practitioner
SP: Social prescribing
NHS: National Health Service
UK: United Kingdom
VCSE: Voluntary, community and social enterprise (organization)

## References

[1] Torjesen I. Social prescribing could help alleviate pressure on GPs. BMJ (Clinical research ed.). 2016;352:i1436. 10.1136/bmj.i1436. 10.1136/bmj.i1436 26965666

[2] Checkland K, Harrison S, McDonald R, Grant S, Campbell S, Guthrie B (2008). Biomedicine, holism and general medical practice: responses to the 2004 general practitioner contract. Sociol Heal Illn.

[3] Polley M, Fleming J, Et A. Making sense of social prescribing. 2017. https://westminsterresearch.westminster.ac.uk/item/q1v77/making-sense-of-social-prescribing. Published 2017. Accessed March 1, 2019. 10.1038/scientificamerican0906-84sp.

[4] Skivington K, Smith M, Chng NR, Mackenzie M, Wyke S, Mercer SW (2018). Delivering a primary care-based social prescribing initiative: a qualitative study of the benefits and challenges. Br J Gen Pract.

[5] Kimberlee R. Developing a social prescribing approach for Bristol. Bristol CCG. 2013; Available from: https://uwe-repository.worktribe.com/output/927254/developing-a-social-prescribing-approach-for-bristol.

[6] Dayson C (2017). Policy commentary - social prescribing “plus”: a model of asset-based collaborative innovation? People. Place and Policy Online.

[7] NHS England. Social prescribing. NHS England. 2019. Available from: https://www.england.nhs.uk/contact-us/privacy-notice/how-we-use-your-information/public-and-partners/social-prescribing/. [cited 2019 Mar 1].

[8] Carter R. NHS England will fund 1,000 social prescribing workers to support practices. Management in Practice. 2019. http://www.pulsetoday.co.uk/news/practice-news-/nhs-england-will-fund-1000-social-prescribing-workers-to-support-practices/20038156.article. Published 2019. Accessed 1 Feb 2019.

[9] Fixsen A, Polley M. Social prescribing for stress related disorders and brain health. In: Clow A, Smyth N, editors. Stress and Brain Health: In Clinical Conditions,Volume 152. Elsevier; 2020. Available from: https://www.elsevier.com/books/stress-and-brain-health-in-clinical-conditions/clow/978-0-12-821116-8. 10.1016/bs.irn.2019.11.005 32450999

[10] Dayson C (2017). Evaluating social innovations and their contribution to social value: the benefits of a “blended value” approach. Policy Polit.

[11] Naylor C, Parsonage M, McDaid D, Knapp M, Fossey M, Galea A. Long-term conditions and mental health: The cost of co-morbidities. King’s Fund Cent Ment Heal. 2012;1–32. Available from: http://www.kingsfund.org.uk/sites/files/kf/field/field_publication_file/long-term-conditions-mental-health-cost-comorbidities-naylor-feb12.pdf.

[12] Caper K, Plunkett J. A very general practice .How much time do GPs spend on issues other than health? Citizens Advice policy briefings Public services in a constrained spending environment. 2015. Available from: https://www.citizensadvice.org.uk/Global/CitizensAdvice/Publicservicespublications/CitizensAdvice_AVeryGeneralPractice_May2015.pdf.

[13] Kimberlee R. What is the value of social prescribing? Adv Soc Sci Res J. 2016;3(3).

[14] Wildman JM, Moffatt S, Steer M, Laing K, Penn L, O’Brien N. Service-users’ perspectives of link worker social prescribing: a qualitative follow-up study. BMC Public Health. 2019. 10.1186/s12889-018-6349-x PMC6341763 30670001

[15] Dixon P (2016). Saving the NHS. J Holist Healthc.

[16] Polley M, Whiteside J, Elnaschie S, Fixsen A. What does successful social prescribing look like? Mapping meaningful outcomes. 2020. National Lottery. Unpublished report. Available at: https://westminsterresearch.westminster.ac.uk/item/qyz67/what-does-successful-social-prescribing-look-like-mapping-meaningful-outcomes.

[17] Social Prescribing Network. The social prescribing landscape. Annu Soc Prescr Netw Conf. 2016;(January):48. Available from: http://www.artshealthresources.org.uk/docs/report-of-the-inaugural-social-prescribing-network-conference/.

[18] Jackson MC (2019). Critical systems thinking and the managment of complexity.

[19] Flood RL, Finnestrand H. A Mighty Step: Critical Systemic Interpretation of the Learning Organization. In: Örtenblad AR, editor. The Oxford Handbook of the Learning Organization. Oxford Books Online; 2019. p. 1–20..

[20] WHO Alliance for Health Policy and Systems. Systems thinking for health systems strengthening. Geneva; 2009. Available from: http://www.who.int/alliance-hpsr/resources/9789241563895/en/.

[21] Whole Systems Partnership (2016). Supporting transformational change – the relational dimension. An introduction to relational value ( R v ) WSP works to support health and social care.

[22] Byass P (2011). Systems thinking for health systems strengthening. Public Health.

[23] Checkland PB (1989). Soft systems methodology. Hum Syst Manag.

[24] Bell S, Christina A (2006). Applying systemic project management approaches for the UK national health service. Syst Pract Action Res.

[25] Churchman CW. Operations Research as a Profession. Manag Sci. 17(2):B37–53.

[26] Ulrich W (1983). Critical heuristics of social planning: a new approach to practical philosophy.

[27] Flood RL (1999). Rethinking the fifth discipline: learning with the unknowable.

[28] Córdoba JR, Midgley G (2006). Broadening the boundaries: an application of critical systems thinking to IS planning in Colombia. J Oper Res Soc.

[29] Bell S, Morse S (2005). Holism and understanding sustainability. Syst Pract Action Res.

[30] Kogetsidis H (2012). Critical Systems Thinking: A Creative Approach to Organizational Change. J Transnatl Manag.

[31] Turner JH (2009). The sociology of emotions: basic theoretical arguments. Emot Rev.

[32] Collins R (2004). Interaction ritual chains.

[33] Shropshire Together. Health and Wellbeing Shropshire. Healthy Lives. 2019. Available from: http://www.shropshiretogether.org.uk/contact-us/. [cited 2019 Mar 28].

[34] Shropshire Food Poverty Allowance. 2019. Available from: https://www.shropshirefoodpoverty.org.uk.

[35] Denzin N, Lincoln Y (2005). The Sage handbook of qualitative research.

[36] Strauss A, Corbin J (2015). Basics of qualitative research: grounded theory procedures and techniques.

[37] Silverman D (2014). Interpreting qualitative data.

[38] Marchand C, Peckham S. Addressing the crisis of GP recruitment and retention: a systematic review. Br J Gen Pract. 2017. 10.3399/bjgp17X689929 PMC5565821 28289014

[39] Flood RL, Romm NRA (2018). A systemic approach to processes of power in learning organizations: part I – literature, theory, and methodology of triple loop learning. Learn Organ.

[40] Ackoff RL (1971). Towards a system of systems concepts. Manag Sci.

[41] Gharajedaghi J (2011). Formulating the Mess. Systems Thinking (Third Edition).

[42] Fixsen A, Ridge D, Evans *C. momma* bear wants to protect”: Vicarious parenting in practitioners working with disturbed and traumatised children. Couns Psychother Res. 2019; Available from: https://onlinelibrary.wiley.com/doi/abs/10.1002/capr.12285.

[43] Seery BL, Corrigall EA (2009). Emotional labor: links to work attitudes and emotional exhaustion. J Manag Psychol.

[44] Bertotti M, Frostick C, Hutt P, Sohanpal R, Carnes D (2018). A realist evaluation of social prescribing: an exploration into the context and mechanisms underpinning a pathway linking primary care with the voluntary sector. Prim Heal Care Res Dev.

[45] Schwarze ML, Bradley CT, Brasel KJ (2010). Surgical “buy-in”: the contractual relationship between surgeons and patients that influences decisions regarding life-supporting therapy. Crit Care Med.

[46] Santoro SL, Martin LJ, Pleatman SI, Hopkin RJ (2016). Stakeholder buy-in and physician education improve adherence to guidelines for Down syndrome. J Pediatr.

[47] Health Improvement Scotland (2019). Community Link Worker initiatives in primary care : key learning from UK studies.

[48] Loftus AM, McCauley F, McCarron MO (2017). Impact of social prescribing on general practice workload and polypharmacy. Public Health.

[49] Midgely G, Midgley G (2000). Boundary critique. Systemic intervention: philosophy, methodology and practice.

[50] Healthy Living Centre Alliance and Scottish Communities for Health and Wellbeing. SPRING Social Prescribing Project : Scotland and Northern Ireland. 2017. https://www.scie.org.uk/transforming-care/innovation/community-based-models/models-of-care-and-support/spring-social-prescribing.

[51] Mercer SW, Fitzpatrick B, Grant L, Chng NR, O’Donnell CA, Mackenzie M (2017). The Glasgow ‘deep end’ links worker study protocol: a quasi-experimental evaluation of a social prescribing intervention for patients with complex needs in areas of high socioeconomic deprivation. J Comorbidity.

[52] Pescheny JV, Pappas Y, Randhawa G (2018). Facilitators and barriers of implementing and delivering social prescribing services: a systematic review. BMC Health Serv Res.

[53] Pescheny J, Randhawa G, Pappas Y. Patient uptake and adherence to social prescribing: a qualitative study. BJGP Open. 2018;2(3). 10.3399/bjgpopen18X101598 PMC6189784 30564731

[54] Mercer SW, Fitzpatrick B, Grant L, Chng NR, McConnachie A, Bakhshi A (2019). Effectiveness of community-links practitioners in areas of high socioeconomic deprivation. Ann Fam Med.

[55] Kleiven HH, Ljunggren B, Solbjør M (2020). Health professionals ’ experiences with the implementation of a digital medication dispenser in home care services – a qualitative study. BMC Health Serv Res.

[56] Doran N, Fox F, Rodham K, Taylor G, Harris M (2016). Lost to the NHS: a mixed methods study of why GPS leave practice early in England. Br J Gen Pract.

[57] Edwards A, Elwyn G (2004). Involving patients in decision making and communicating risk: a longitudinal evaluation of doctors’ attitudes, and confidence during a randomized trial. J Eval Clin Pract.

[58] Polley M. Core principles of social prescribing and its impact on health and wellbeing. Media Central UCL. 2018; Available from: https://mediacentral.ucl.ac.uk/Play/12838.

