# Supplementary material for: Applying critical systems thinking to social prescribing: a relational model of stakeholder “buy-in”
Source: BMC Health Serv Res. 2020 Jun 24;20:580. doi: 10.1186/s12913-020-05443-8 (PMC7312116; doi:10.1186/s12913-020-05443-8)
Supplement: Supplementary file 1 — Additional file 1. Service user interview schedule: List of open questions used as a guide during the semi-structured interviews with service users for the qualitative arm of the service evaluation Shropshire social prescribing project. [file 12913_2020_5443_MOESM1_ESM.docx]

**Service Users Interview Schedule: Shropshire project**

1. Are you familiar with the term social prescribing and if so what does it mean to you?

- (explain in simply terms what it is and how it connects with their visit to a link worker)

1. Can you tell me something about how you came to be aware or got referred into the Social Prescribing Service?
2. What was your experience of your initial assessment with the social prescribing Link worker (Community Advisor)?
3. Can you explain what happened after the initial assessment?
4. To what extent were you able to link in with a service to support your needs?
   1. Probes- access, communication, feedback
5. After your first meeting, what kind of contact did you have with the Social prescribing link worker/ Advisor?
6. What differences, if any, has the Social Prescribing Service made to you?
   1. Probes- being part of group, health, family, visits to GPs?
7. What challenges if any have you encountered in carrying through the action plan?
8. Are there any recommendations you would make about improving the service?
9. What are your overall impressions of social prescribing?
