# Supplementary material for: Applying critical systems thinking to social prescribing: a relational model of stakeholder “buy-in”
Source: BMC Health Serv Res. 2020 Jun 24;20:580. doi: 10.1186/s12913-020-05443-8 (PMC7312116; doi:10.1186/s12913-020-05443-8)
Supplement: Supplementary file 2 — Additional file 2. Professionals interview schedule: List of open questions used as a guide during the semi-structured interviews with professionals for the qualitative arm of the service evaluation Shropshire social prescribing project. [file 12913_2020_5443_MOESM2_ESM.docx]

**Professionals Interview schedule: Shropshire social prescribing project**

1. Tell me about your background and present work.
2. Tell me about your experiences of the social prescribing service.

- Defining it (what is the service – specific remit)
- Differentiating it (How is it different from other services)
- How it works alongside existing services.
- Where is sits within the context of Public Health/Local Authority (Managing relationships)

1. (where relevant).Tell me about your experiences of setting up and running the evaluation.
2. What so far have been some of the challenges of setting up/ referring people into the existing service?
3. What so far have been the benefits to staff and patients (in your experience)?
4. Is there anything you would recommend to others who are setting up similar schemes?
