# Supplementary material for: Applying critical systems thinking to social prescribing: a relational model of stakeholder “buy-in”
Source: BMC Health Serv Res. 2020 Jun 24;20:580. doi: 10.1186/s12913-020-05443-8 (PMC7312116; doi:10.1186/s12913-020-05443-8)
Supplement: Supplementary file 3 — Additional file 3. Word frequency cloud core stakeholder interviews: created through NVivo software, this diagram illustrates the frequency with which participants who were core team members used a word or phrase during the semi-structured interview. The word at the “heart” of the cloud is “people,” pointing to the importance placed on the relational element of social prescribing. [file 12913_2020_5443_MOESM3_ESM.pdf]

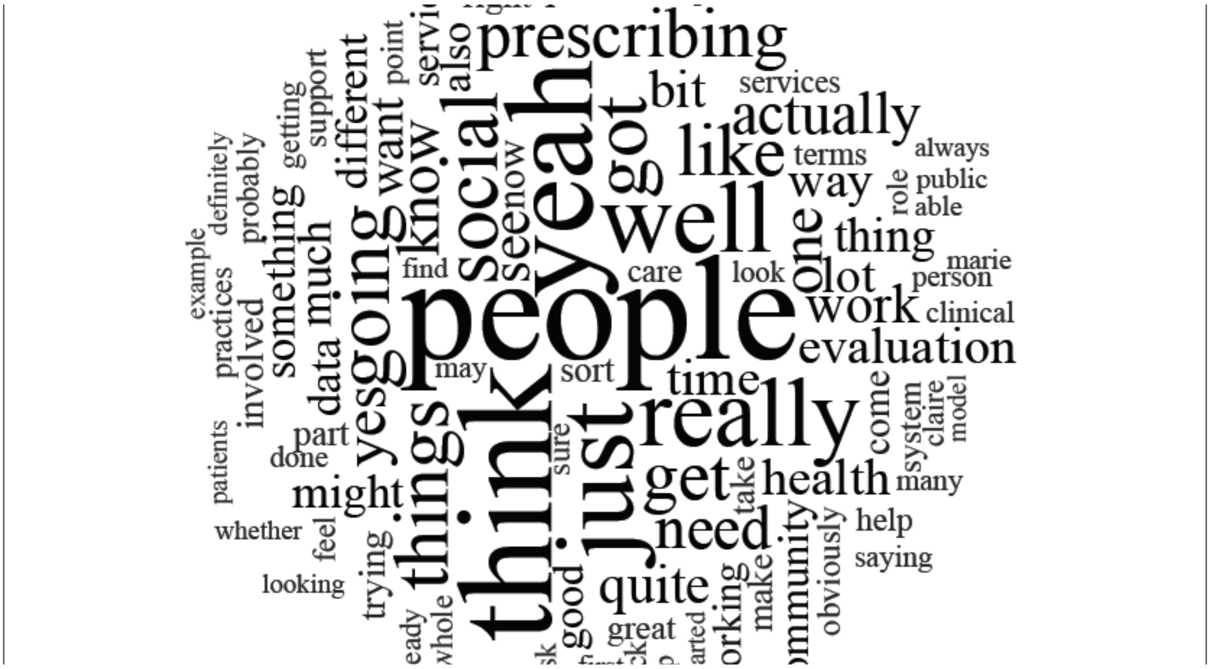
people
think
yeah
prescribing
well
really
just
social
going
things
like
actually
got
know
need
get
quite
health
evaluation
work
lot
thing
one
way
time
bit
yes
might
something
much
data
different
want
also
see
now
find
may
sort
care
look
services
terms
always
public
role
able
marie
person
clinical
come
system
claire
model
many
help
saying
obviously
make
take
sure
good
great
part
done
feel
trying
whole
looking
whether
patients
involved
practices
example
definitely
probably
getting
support
point
